# The Role of Exerkines in Obesity-Induced Disruption of Mitochondrial Homeostasis in Thermogenic Fat

**DOI:** 10.3390/metabo14050287

**Published:** 2024-05-17

**Authors:** Hui Shao, Huijie Zhang, Dandan Jia

**Affiliations:** 1 School of Exercise and Health, Shanghai University of Sport, Shanghai 200438, China; shaohui@hrbipe.edu.cn (H.S.); 2321518039@sus.edu.cn (H.Z.); 2 Graduate School of Harbin Sport University, Harbin Sport University, Harbin 150006, China

**Keywords:** obesity-associated metabolic disorder, thermogenic fat, mitochondrial homeostasis, exerkines

## Abstract

There is a notable correlation between mitochondrial homeostasis and metabolic disruption. In this review, we report that obesity-induced disruption of mitochondrial homeostasis adversely affects lipid metabolism, adipocyte differentiation, oxidative capacity, inflammation, insulin sensitivity, and thermogenesis in thermogenic fat. Elevating mitochondrial homeostasis in thermogenic fat emerges as a promising avenue for developing treatments for metabolic diseases, including enhanced mitochondrial function, mitophagy, mitochondrial uncoupling, and mitochondrial biogenesis. The exerkines (e.g., myokines, adipokines, batokines) released during exercise have the potential to ameliorate mitochondrial homeostasis, improve glucose and lipid metabolism, and stimulate fat browning and thermogenesis as a defense against obesity-associated metabolic diseases. This comprehensive review focuses on the manifold benefits of exercise-induced exerkines, particularly emphasizing their influence on mitochondrial homeostasis and fat thermogenesis in the context of metabolic disorders associated with obesity.

## 1. Introduction

Obesity has evolved into a pervasive global epidemic, with its prevalence steadily increasing on a worldwide scale [1]. According to estimates from the World Health Organization (WHO), approximately 13% of adults globally grapple with obesity. Importantly, obesity serves as a precursor to metabolic syndrome, giving rise to a spectrum of complications, including but not limited to diabetes, hypertension, non-alcoholic fatty liver disease (NASH), cardiovascular disorders, neuropathic diseases, and cancer [2,3,4,5,6]. In the COVID-19 pandemic, the increased mortality in patients with obesity is a noteworthy example. Hence, there is urgent need for effective treatment methods to prevent and mitigate obesity-associated metabolic disorders and their related complications.

Mitochondria play a pivotal role in preserving energy metabolism in adipose tissues. However, obesity leads to the pathological remodeling of mitochondrial morphology and dysfunction in adipocytes [7,8]. Dysfunction of mitochondria has adverse effects on glucose and lipid metabolism, oxidative capacity, insulin sensitivity, adipocyte differentiation, and thermogenesis in adipocytes, ultimately contributing to metabolic diseases [8,9,10]. Enhancing mitochondrial function, achievable through various approaches such as mitochondria-targeted antioxidants, thiazolidinedione, dietary natural compounds, controlled caloric restriction, and regular exercise, plays a crucial role in maintaining metabolic homeostasis [11,12]. This contribution is evidenced by the promotion of thermogenesis in brown and beige adipocytes. As a secure, effective, and cost-efficient approach, regular exercise is widely embraced by individuals dealing with obesity and overweight [13,14]. Exerkines, induced by exercise, play pivotal roles in maintaining mitochondrial homeostasis, facilitating fat browning and thermogenesis as a defense against obesity-associated metabolic diseases [15,16]. In this review, our emphasis is on elucidating the advantages of regular exercise concerning fat thermogenesis and mitochondrial homeostasis in the context of metabolic diseases associated with obesity. We explore the role of exercise in stimulating the secretion of exerkines and its potential significance in preventing obesity-associated metabolic disorders.

## 2. Mitochondrial Homeostasis in Thermogenic Fat

**Figure 1:**
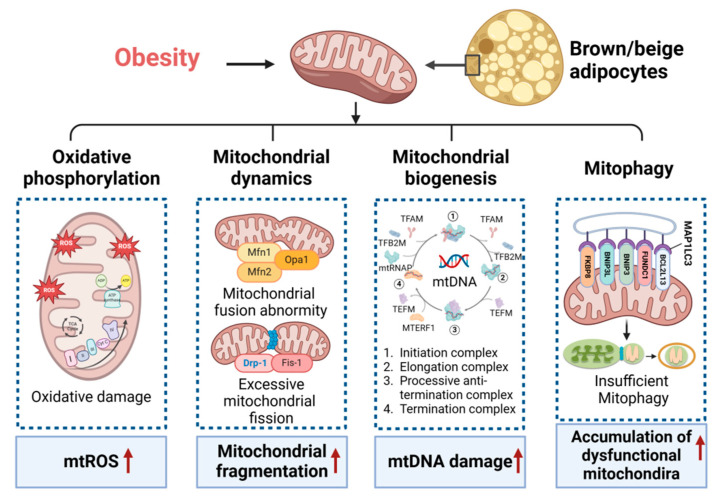
Mitochondrial homeostasis in thermogenesis fat. Obesity results in the disruption of mitochondrial homeostasis in thermogenesis fat, affecting mitochondrial oxidative phosphorylation, dynamics, biogenesis, and mitophagy. This is evidenced by the increased mitochondrial reactive oxygen species (mtROS), mitochondrial fragmentation, mitochondrial DNA (mtDNA) damage, and the accumulation of dysfunctional mitochondria. Mfn1/2, mitofusin 1 and 2; Opa1, optic atrophy 1; Drp-1, dynamin-related protein 1; Fis-1, mitochondrial fission protein 1; TFAM, mitochondrial transcription factor A; TFB2M, mitochondrial transcription factors B2; mtRNAP, mitochondrial RNA polymerase; TEFM, transcription elongation factor of mitochondria; MTERF1, mitochondrial transcription termination factor 1; MAPP1LC3, microtubule-associated protein 1 light chain 3; BCL2L13, Bcl-2-like protein 13; FUNDC1, FUN14 domain-containing protein 1; BNIP3, BCL2 interacting protein 3; FKBP8, FK506 binding protein 8. Red arrows indicate an increase (up arrow).

Mitochondrial homeostasis encompasses the balance and regulation of various processes within mitochondria, including mitochondrial oxidative phosphorylation, mitochondrial dynamics, mitochondrial biogenesis, mitophagy, and mitochondrial uncoupling. This delicate equilibrium plays a pivotal role in sustaining energy metabolism, particularly in brown and beige adipocytes, distinguished by a heightened abundance of mitochondria. Disruption of mitochondrial homeostasis results in adverse effects on lipid metabolism, adipocyte differentiation, oxidative capacity, inflammation, insulin sensitivity, and thermogenesis, culminating in metabolic diseases [8,15,17,18]. Enhancing mitochondrial homeostasis in thermogenic fat emerges as a potential avenue for developing treatments for metabolic diseases (Figure 1).

### 2.1. Mitochondrial Function in Thermogenic Fat

Mitochondria play a crucial role in metabolism of adipose tissue, as evidenced by their involvement in crucial metabolic pathways, such as lipolysis and lipogenesis. These functions of mitochondrial are essential for supporting energy metabolism in thermogenic adipocytes. Dysfunction of mitochondrial function in brown and beige adipocytes is associated with disrupted thermogenesis and energy balance in obesity and aging. Given that the metabolism of thermogenic fat is primarily oxidative, the effective regulation of thermogenesis in these cells involved manipulating the rate-limiting steps in mitochondrial respiration and oxidative phosphorylation [9]. Furthermore, recent studies have revealed that mitochondria can be exchanged between cells, such as adipocytes and macrophages, to regulate metabolism, homeostasis, and thermogenesis in brown and beige adipocytes. This exchange is facilitated through the release of extracellular vesicles (EVs) carrying oxidatively damaged mitochondrial components, thereby preventing the breakdown of the thermogenic program [7,19,20]. Additionally, mitochondria-derived EVs have been shown to decrease the expression of peroxisome proliferator-activated receptor-γ and uncoupling protein 1 (UCP1). Phospholipid cardiolipin (CL) and phosphatidic acid (PA) play pivotal roles in regulating mitochondrial morphology and mitochondrial function [21]. Lipocalin 2, a protein that binds to PA, assumes a crucial role in the remodeling of acyl-chain remodeling in phospholipids and in regulating mitochondrial function within thermogenic fat. This process is particularly significant during inflammation induced by obesity and metabolic stimulation resulting from cellular aging [22].

A recent study has indicated that there is a sex-based difference in the impact on adipose mitochondrial function and the development of metabolic syndrome. In females, adipose mitochondrial function shows heightened activity, encompassing elevated mitochondrial oxidative phosphorylation, mitochondrial DNA content, and augmented production of mitochondrial reactive oxygen species. These factors are closely linked to adiposity, insulin resistance, and plasma lipid levels [23]. Several studies have underscored the pivotal role of mitochondrial metabolism in the pro-inflammatory activation of adipose tissue macrophages in response to obesity [7,8]. The application of a near-infrared fluorophore with a preferential accumulation in the mitochondria of adipose tissue macrophages has been shown to mitigate pro-inflammatory activation by enhancing the levels of mitochondrial complex and oxidative phosphorylation [8]. Additionally, the respiratory chains of mitochondria in interscapular brown adipose tissue depend on UCP1 [24].

### 2.2. Mitochondrial Biogenesis in Obesity

Previous studies have suggested that obesity leads to a decline in mitochondrial biogenesis, a reduction in the expression of genes responsible for mitochondrial respiratory complex components, and a decrease in respiration/mitochondrial oxidative phosphorylation (OXPHOS) in adipose tissue [25,26]. Specifically, the cardiotrophin-like cytokine factor 1 (CLCF1) has been identified as a key player in inducing the whitening of brown adipose tissue and impeding thermogenesis. This effect is achieved by suppressing mitochondrial biogenesis through the activation of the STAT3/PGC1α signaling pathway in response to obesity [26]. It is important to observe that enhancing mitochondrial biogenesis is critical for addressing metabolic diseases associated with obesity.

A recent study has identified that the thyroid hormone triiodothyronine (T_3_) triggers thermogenesis by uncoupling electron transport from ATP synthesis in BAT mitochondria. T_3_ enhances fatty acid oxidation, autophagic flux, mitophagy, mitochondrial respiration, and mitochondrial biogenesis [27]. Additionally, Parkin plays a role in maintaining mitochondrial homeostasis in white adipocytes by orchestrating a balance between mitophagy and Pgc1alpha-mediated mitochondrial biogenesis. This suggests a promising therapeutic target within adipocytes to address obesity and obesity-associated disorders. CL, a phospholipid located in the inner membrane of mitochondria, exerts a major role in maintaining mitochondrial metabolism and structural integrity. It is essential for the well-being of various organs, including fat, liver, heart, skeletal muscle, brain, and kidney [28,29,30,31,32]. Furthermore, CL acts as a pivotal regulator in the thermogenic programs, connecting with mitochondrial biogenesis and function in response to regular exercise [15]. Lifelong exercise has been identified as a beneficial factor, mitigating age-related changes in mitochondrial biogenesis, inflammation, and lipolysis in perirenal fat and liver tissues. This positive impact may involve the inhibition of inflammation through activating the c-Jun N-terminal kinase (JNK), p38 mitogen-activated protein kinase (MAPK), and AKT pathways in adipose tissue [33].

### 2.3. Mitophagy in Thermogenic Fat

Autophagy is necessary for the efficient turnover of damaged organelles, including mitochondria (mitophagy). Mitophagy refers to a selective process in which damaged mitochondria are isolated and subsequently eliminated through autophagic degradation [34]. The adipose-specific deletion of autophagy-related 7 results in a large volume of cytosol and contained more mitochondria in mutant white adipocytes [35]. Furthermore, exposure to cold induces adaptive thermogenesis by enhancing the autophagy of lipids and mitophagy in brown and beige adipocytes [36,37]. Recent studies have revealed that T_3_ not only regulates mitochondrial homeostasis by inducing lipophagy and mitophagy in the liver and skeletal muscle [38,39], but also triggers thermogenesis. This involved inducing the expression of mitochondrial UCP1, promoting autophagy-dependent fatty acid oxidation, and regulating autophagy, activity, and turnover of mitochondria in BAT and aging skeletal muscle [27,40]. In contrast, BAT primarily relies on upregulated mitophagy and mitochondrial biogenesis to ensure mitochondrial quality control. Consequently, promoting autophagy to induce mitochondrial turnover in BAT could hold therapeutic potential for enhancing thermogenesis and addressing obesity and associated metabolic conditions.

Nevertheless, diminished mitophagy may also prove essential in the browning of white adipose, allowing for a substantial increase in mitochondrial mass during this remodeling process. Furthermore, even with the suppression of p62 and optineurin, rosiglitazone continued to promote UCP1 expression, endorsing the idea that a reduction in mitophagy machinery facilitates beige remodeling [41]. Deficiency in FUNDC1, a mediator of mitophagy, triggered a retrograde response in muscle, leading to the upregulation of fibroblast growth factor 21 (FGF21) expression. This, in turn, facilitated the thermogenic remodeling of adipose tissue in response to obesity [42]. The tumor suppressor p53 enhances insulin sensitivity in aged adipose tissue by triggering mitophagy [43].

### 2.4. Mitochondrial Uncoupling in Obesity

Mitochondrial uncoupling is characterized by a dissociation between the generation of mitochondrial membrane potential and its utilization for ATP synthesis, a process vital for mitochondria-dependent energy production. Recent research has revealed that mitochondrial uncoupling extends beyond its association with mitochondrial dysfunction and is also implicated in various biological processes, including the production of ROS, autophagy, cell death, protein secretion, and metabolic adaptation in brown and beige adipocytes [44]. The subtle uncoupling of oxidative phosphorylation achieved through numerous mitochondria-targeted penetrating cations plays a role in the reported therapeutic benefits by inducing autophagy and mitophagy [45]. The initiation of mitochondrial uncoupling, whether through synthetic or natural uncoupling agents or by activating UCPs, sets in motion multiple cellular mechanisms [46,47].

Mitochondria uncoupling can serve a dual role, offering protection against cell death and apoptosis in certain instances while potentially promoting them, contingent upon factors such as cell type, the specific mitochondrial uncoupler employed, and the intensity of mitochondrial uncoupling [48,49]. The mitochondrial uncouplers like carbonyl cyanide p-trifluoromethoxyphenylhydrazone (FCCP) or 2,4-dinitrophenol (DNP) may disturb the equilibrium of various, including Ca^2+^, Na^+^, and K^+^ at cytosolic, mitochondrial, or lysosomal levels [47,50,51]. A recently identified mitochondrial uncoupler, BAM15, improves body fat mass, inflammation, and insulin resistance in obese mice [49]. The recognition of UCP1′s involvement is pivotal in understanding the thermogenic processes within brown and beige adipocytes [52]. More recently, thermogenic processes that are independent of UCP1 have been observed in thermogenic fat [53,54].

## 3. The Impact of Exercise on Thermogenic Fat

### 3.1. Exercise-Induced Browning of White Adipocytes

**Figure 2:**
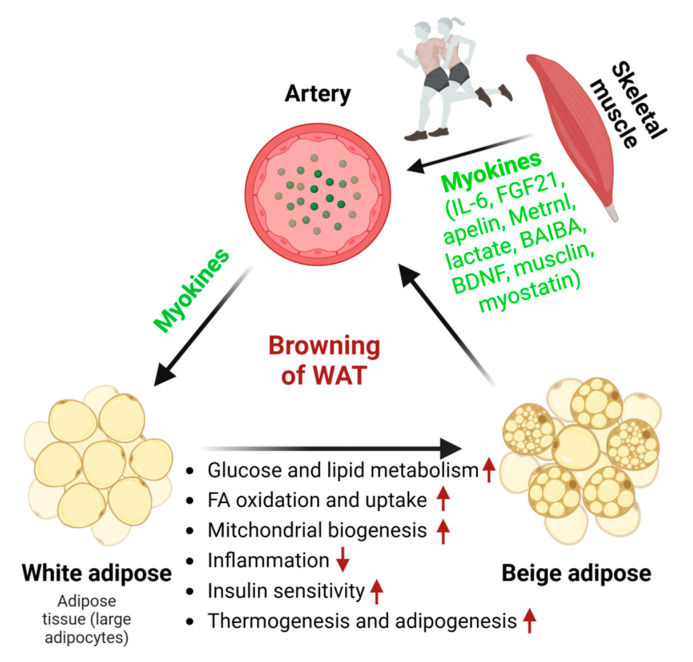
The effect of myokines on white adipose tissue browning. Exercise training promotes the browning of WAT by triggering the secretion of various myokines from skeletal muscle. This is substantiated by reductions in adipocyte size, heightened lipid and glucose metabolism, increased FFA oxidation and uptake, enhanced mitochondrial biogenesis, improved insulin sensitivity, brown adipogenesis, and thermogenesis. Myokines are secreted from skeletal muscle in response to exercise. Red arrows indicate an increase (up arrow) or a decrease (down arrow).

Consistent exercise training (e.g., 11 days of wheel cage running, swim training performed for 90 min daily, 5 days/week) substantially reduces adipocyte size, enhances mitochondrial biogenesis and glucose uptake, stimulates adipokine secretion, and improves overall metabolic health in WAT [55,56]. Four-week wheel-running exercise training dramatically curtails body weight gain, promotes energy expenditure, and increases UCP1-dependent thermogenesis [57]. Prolonged treadmill-running exercise training (3 m/min for 5 min, increased to 4.8–5 m/min for 5 min, and then reaching a maximum of 7.2–8 m/min for 20 min; 0% slope) induces adaptability in white adipose depots, as demonstrated by increasing free fatty acid (FFA) oxidation, reduced inflammation, and diminished macrophage infiltration in aged obese female mice [58]. Prolonged exercise (e.g., treadmill with 10 m/min for the first 60 min, followed by 1 m/min increment increases at 15-min intervals or treadmill with a fixed 10% slope at a constant 18 m/min speed for 60 min daily for 5 d/wk (8 wk) before test) leads to mitochondrial biogenesis and beiging in WAT by regulating myokines, including IL-6, FGF21, apelin, meteorin-like protein (Metrnl), lactate, beta-aminoisobutyric acid (BAIBA), brain-derived neurotrophic factor (BDNF), musclin, myostatin, and irisin [59,60,61,62]. The activation of the Wnt/β-catenin signaling pathway and PGC-1α-related pathways drives the adipocyte population necessary for beiging, which is involved in mitochondrial biogenesis and function [63] (Figure 2).

### 3.2. Exercise Modulates Brown Adipose Tissue

The efficacy of BAT has been reported to be blunted by metabolic diseases [64], cardiovascular diseases [65], and aging [66,67,68,69]. While traditionally recognized as a thermogenic tissue, BAT communicates with distant organs, such as the heart, through its endocrine function. Remarkably, four weeks of swimming exercise (90 min twice per day) induce the release of the small extracellular vesicles (sEVs) from BAT, conferring cardioprotection by delivering the cardioprotective miRNAs to the heart during myocardial ischemia/reperfusion (MI/R) injury [65]. Meanwhile, the augmented exercise capacity induced by BAT is mediated through mitochondrial biogenesis, antioxidant defense, and enhanced hindlimb perfusion. Therefore, BAT serves as a mediator for heightened exercise capacity, a mechanism further potentiated by the disruption of the regulator of G protein signaling 14 (RGS14) [70]. Mitochondrial homeostasis in BAT plays a pivotal role in the thermoregulatory and metabolic processes. Voluntary physical exercise promotes thermogenesis, insulin sensitivity, mitochondrial activity, and biogenesis in BAT [70,71,72]. CL is a key effector of brown/beige adipocytes’ thermogenic programs and is linked to mitochondrial biogenesis and function in response to regular exercise [15].

### 3.3. Exercise-Induced UCP1-Dependent Thermogenesis

Situated in the inner membrane of mitochondria, UCP1 induces a proton leak across this membrane, facilitating the conversion of electrochemical energy into heat. Notably, mice lacking UCP1 exhibit impaired thermogenesis, underscoring the pivotal role of UCP1 in nonshivering heat production. Given that UCP1 is a key regulator in the thermogenesis of brown adipocytes and a subset of white adipocytes, various other functional thermogenic elements exert their impacts in a UCP1-dependent manner. Beige adipocytes expressing UCP1 can be activated through exposure to cold, administration of β-adrenergic agonists, or engagement in exercise training to stimulate thermogenesis [55,73,74].

Exercise training is said to confer benefits, at least in part, by enhancing mitochondrial uncoupling-driven thermogenesis. The global stimulation of mitochondrial uncoupling by exercise training contributes to the restructuring of skeletal muscle cell physiology. Furthermore, acute physical exercise training results in an upregulation of BAT UCP1 protein expression in individuals with obesity [75]. The immediate impact of a single exercise session on thermogenesis can be elucidated through the increase in leptin-induced hypothalamic ERK1/2 phosphorylation. Indeed, a single exercise session elevates hypothalamic sphingine-1-phosphate (S1P) levels and STAT3 phosphorylation events that ultimately enhance UCP1-dependent BAT thermogenesis [76,77]. In WAT, the impact of exercise training appears to be contrary. Specifically, exercise training (17 m/min, 45 min/day, 5 days, 8 weeks) is observed to diminish the protein expression of UCP1 and PGC-1α in the subcutaneous WAT of mice subjected to a high-fat diet [78]. Additionally, exercise training triggers the release of myokines by skeletal muscle. Among these myokines, irisin holds particular significance. Irisin’s primary and extensively studied role is to instigate the browning of WAT, consequently promoting UCP-1-dependent mitochondrial uncoupling [79].

### 3.4. Exercise-Induced UCP1-Independent Thermogenesis

UCP1 has significantly advanced our comprehension of how these cells participate in thermogenesis [52]. However, cold-acclimated *Ucp1* knockout mice still display tolerance to cold exposure, suggesting the presence of compensatory thermogenic mechanisms [80]. Subsequently, thermogenic processes independent of UCP1 have been elucidated, both within thermogenic fat and in other tissues. A recent study has identified several UCP1-independent thermogenic effectors using *Ucp1* knockout mice, including creatine, Dio2, calcium-ATPase, glycerol-3-phosphate shuttle, PGC-1α, and Cox II [81].

**Figure 3:**
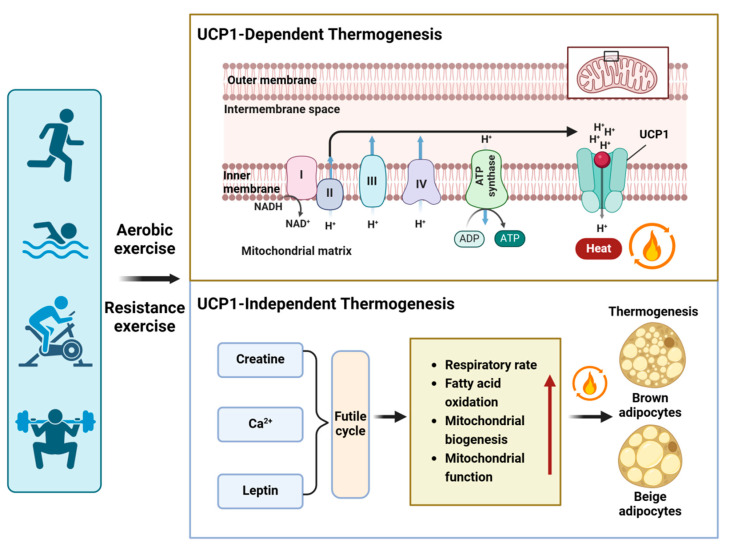
Exercise-induced thermogenesis in adipocytes. Exercise not only activates UCP1-dependent thermogenesis but also induces UCP1-independent thermogenesis by increasing various futile cycles, such as creatine futile cycling, Ca^2+^ futile cycling, and leptin-induced TAG-fatty acid cycling. The mechanisms of UCP1-independent thermogenesis involve respiratory rate, fatty acid oxidation, mitochondrial biogenesis, and function. Red arrows indicate an increase (up arrow).

The voluntary wheel running exercise can mitigate cold-induced weight loss, and in this process, UCP-1 does not appear to play a role [82]. Muscle, functioning as a thermogenic organ, actively contributes to maintaining body temperature in cold conditions. Sarcolipin (SLN), which uncouples calcium transport from adenosine triphosphate hydrolysis by sarco/endoplasmic reticulum Ca^2+^-ATPase (SERCA), has been proposed as a potential mechanism for oxidative metabolism and nonshivering thermogenesis in skeletal muscle [83,84,85,86]. A recent study has provided additional evidence that Ca^2+^ cycling plays a regulatory role in thermogenesis within beige adipocytes and contributes to overall energy homeostasis in the body [87]. In *Ucp1* knockout mice, alterations in Ca^2+^ cycling were observed, as evidenced by the increased fatty acid oxidation. These findings suggest that Ca^2+^ cycling plays a role in Ucp1-independent thermogenesis in WAT. Furthermore, the study indicates that myokine (leptin) triggers thermogenesis through a UCP1-independent mechanism involving futile substrate cycling [88,89,90] (Figure 3).

## 4. Potential Impact of Exerkines on Mitochondrial Homeostasis in Thermogenic Fat

Emerging evidence suggests that regular exercise is a widely recognized therapeutic tool and highly effective intervention for mitigating obesity-associated metabolic syndrome. It plays a crucial role in mitochondrial homeostasis as well as contributing significantly to individual thermogenic activity [65,91,92,93,94,95]. Exercise-induced circulating factors, referred to as exerkines, are implicated in the activation and metabolism of BAT and promote the browning of WAT [65,96,97,98]. Exercise induces the secretion of exerkines from various tissues, including skeletal muscle (myokines), white adipose tissue (adipokines), and brown adipose tissue (batokines).

### 4.1. The Impact of Myokines on Mitochondrial Homeostasis

Exercise has been recognized as a therapeutic approach for managing obesity-related metabolic diseases by mitigating abdominal adiposity and metabolic syndrome. Despite this acknowledgement, the underlying mechanisms of how regular exercise training serves as a therapeutic modality for abdominal fat remain rudimentary. Exercise-induced myokines have the potential to stimulate the browning of WAT by modulating lipid metabolism in response to obesity-associated metabolic diseases. Notably, the myokines involved include IL-6 [99], FGF21 [100], apelin [101], Meteorin-like (Metrnl) [102,103,104], lactate [105], β-aminoisobutyric acid (BAIBA) [106,107], BDNF [108], musclin [109], myostatin [16], and irisin-an exercise-induced myokine dependence PGC-1α [79] (Table 1).

### 4.2. The Impact of Adipokines on Mitochondrial Homeostasis

**Figure 4:**
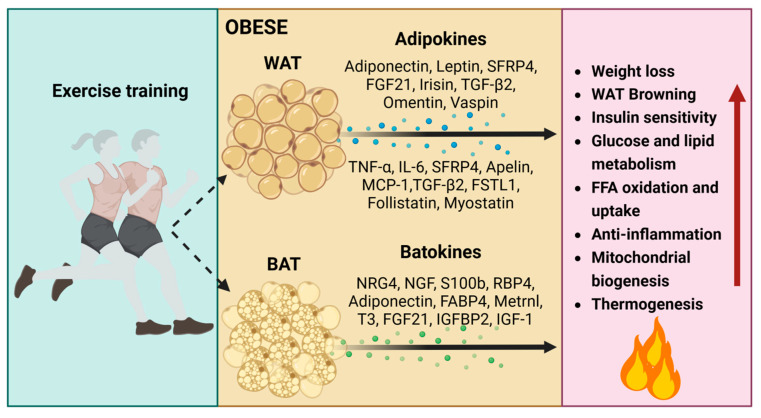
The impacts of regular exercise-induced adipokines and batokines on obesity. Adipokines and batokines induced by regular exercise play crucial roles in influencing glucose tolerance, fatty acid metabolism, insulin sensitivity, inflammation, mitochondrial homeostasis, the sympathetic neural network, as well as thermogenesis in both BAT and WAT. T3, thyroid hormone; IGFBP2, insulin-like growth factor binding protein 2; IGF-1, insulin-like growth factor 1. Red arrows indicate an increase (up arrow).

Adipokines secreted by exercise-trained white adipose tissue: their endocrine effects on enhanced glucose tolerance, fatty acid metabolism, and insulin sensitivity. Noteworthy, adipokines include adiponectin, leptin, SFRP4, FGF21, TGF-β2, follistatin-like 1, omentin, and vaspin [111,113,115,116,117,126,127,128,129,130]. These contribute to the reduction in inflammation through adipokines like SFRP5, TNF-α, IL-6, Wnt family member 5A, and MCP-1 [74,131,132,133]. Moreover, exercise-trained WAT promotes the emergence of thermogenic brown-like adipocytes with adipokines such as apelin, TGF-β, follistatin, and myostatin [101,114,116]. This collective action not only supports mitochondrial homeostasis but also enhances overall metabolic homeostasis in response to exercise [56,79,102,128,134,135,136] (Figure 4, Table 1).

A comprehensive meta-analysis study revealed that exercise, coupled with dietary intervention, significantly modulates adiponectin and leptin levels in individuals who are overweight or obese [126]. Adiponectin regulates mitochondrial biogenesis and insulin resistance in individuals with obesity subjected to treadmill running exercise [115]. The exercise-responsive transcript, SFRP4, emerges as a key mediator in facilitating long-term exercise-induced enhancements in insulin resistance [129]. Additionally, TGF-β2, an exercise-induced adipokine, mediates glucose homeostasis, improves insulin sensitivity, increases FFA uptake and oxidation, and promotes mitochondrial function in response to diabetes [113,137]. FSTL1 plays a significant role in inflammation, glucose metabolism, and insulin sensitivity, particularly in the context of obesity and exercise [116]. A 12-week aerobic exercise training increases the expression of adipokine omentin in visceral AT, leading to the regulation of insulin sensitivity, glucose homeostasis, and anti-inflammatory effects against type 2 diabetes mellitus [117]. Omentin also serves as a positive regulator of mitochondrial biogenesis by activating AMPK-PGC1alpha pathway [138]. Prolonged exercise treatment dramatically upregulates apelin, a key contributor to the presence of thermogenic brown-like adipocytes. This elevation is associated with increased glucose uptake, mitochondrial biogenesis, and decreased insulin resistance in individuals with diabetes [101,139,140]. Additionally, aerobic exercise training induces the activation of the fat browning-related pathway (AMPK/Sirt1/PGC-1α), improves free fatty acid oxidation, reduces inflammation, promotes mitochondrial biogenesis, and facilitates the synthesis and secretion of exerkines, including irisin, TNF-α, IL-10, and MCP-1 [62,141,142] (Table 1)

### 4.3. The Impact of Batokines on Mitochondrial Homeostasis

Regular exercise-induced sympathetic nervous system (SNS) plays a significant role in the thermogenesis of brown/beige adipocytes and the maintenance of mitochondrial homeostasis by regulating the release of neurotrophic batokines [143,144,145]. These batokines contribute to the remodeling of the sympathetic neural network and the promotion of thermogenesis, including neuregulin-4 (NRG4) [146,147,148], nerve growth factor (NGF) [149,150], and calcium-binding protein B (S100b) [143,151,152] (Figure 4). The neurotrophic batokines further stimulate the formation of brown/beige adipocytes and enhance mitochondrial biogenesis by uncoupling oxidative phosphorylation from ATP production.

NRG4 mitigates the onset of obesity and fosters metabolic well-being by increasing BAT thermogenic activity in response to exercise (e.g., high-intensity interval training, circuit resistance training). This is substantiated by an upregulation in the expression of thermogenic markers (UCP1 and PRDM16), a decrease in the expression of lipogenic/adipogenic genes (*Pparγ* and *Cd36*), an increase in the number of brite/beige adipocytes, neurite outgrowth, blood vessels, and improvements in glucose homeostasis and mitochondrial homeostasis within adipose tissues [148,153,154,155]. S100b, on the other hand, stimulates neurite production and addresses deficient sympathetic innervation caused by Calsyntenin-3β deficiency, a mammal-specific endoplasmic reticulum membrane protein. This deficiency is implicated in functional sympathetic innervation and the maintenance of mitochondrial homeostasis in adipose tissues as a countermeasure against obesity [143,151,156]. Nevertheless, the accumulation of oxidative stress-induced S100b promotes the transition of myoblasts into brown adipocytes. This is substantiated by the increased expression of PRDM16 [157,158], the upregulation of bone morphogenetic protein 7 (BMP-7), which enhances differentiation of brown preadipocytes, and the promotion of mitochondrial biogenesis [159]. Furthermore, hormones are released from BAT in response to cyclic AMP–mediated thermogenic activation. These hormones include adiponectin, adipsin, fatty acid–binding protein 4 (FABP4), retinol-binding protein 4 (RBP4), chemerin, clusterin, and macrophage migration inhibitory factor [148] (Figure 4)

## 5. Conclusions

Disruption of mitochondrial homeostasis induced by obesity has adverse effects on lipid metabolism, adipocyte differentiation, oxidative capacity, inflammation, insulin sensitivity, and thermogenesis. Elevating mitochondrial homeostasis in thermogenic fat emerges as a promising avenue for developing treatments for metabolic diseases. The exerkines (myokines, adipokines, batokines) released during exercise have the potential to improve glucose and lipid metabolism, ameliorate mitochondrial homeostasis, and stimulate fat browning and thermogenesis in response to obesity-associated metabolic diseases. A thorough comprehension of the intricate interplay between mitochondrial homeostasis and thermogenesis in adipose tissue, along with the advantageous effects of exercise, could lead to the development of non-pharmacological therapeutic strategies to prevent obesity-related metabolic diseases.

## Figures and Tables

**Table 1:** The benefits of exerkines in obesity-associated metabolic diseases.

Exerkines	Main Mechanism	Main Biological Action	Main Target Tissue	Refs.
Lactate	Lactate/AMPK/ SIRT1/PGC-1α	Induces adipose browning, increases mitochondrial biogenesis and thermogenesis	AT, Skeletal muscle	[110]
IL-6	IL-6/STAT3/ AMPK	Induces weight loss, alleviates obesity-induced fatty liver and insulin resistance	AT, Liver	[99]
FGF21	FGF21/Adiponectin/ERK	Regulates glucose and lipid homeostasis, alleviates hyperglycemia and insulin resistance	AT	[111]
Apelin	Apelin/PRDM16	Promotes brown adipogenesis and thermogenesis, prevents metabolic dysfunction	BAT	[101]
Metrnl	Metrnl/PGC-1α/ PI3K/Akt/NF-κB	Regulates energy expenditure, promotes beige fat thermogenesis and anti-inflammatory	AT	[102]
BAIBA	BAIBA/PGC-1α/PPARα	Induces browning of WAT, and promotes glucose homeostasis and β-oxidation	WAT	[107]
BDNF	BDNF/Adiponectin/CD80	Regulates inflammatory profile and arterial thrombosis	WAT	[108]
Musclin	Musclin/GLUT-4	Improves lipid metabolism and insulin sensitivity	Skeletal muscle	[112]
TGF-β2	Lactate/TGF-β2	Promotes glucose and fatty acid metabolism	AT	[113]
Myostatin	Follistatin/Myostatin/TGF-β	Promotes adipose browning and increases mitochondrial biogenesis	AT	[114]
Irisin	Irisin/PGC-1α	Increases UCP1 expression, promotes brown-fat-like development	WAT	[79]
Adiponectin	APPL1/SIRT1/ PGC-1α	Regulates mitochondrial biogenesis, improves insulin resistance	Skeletal muscle	[115]
FSTL1	FSTL1/Apelin	Increases glucose metabolism and insulin sensitivity	Skeletal muscle	[116]
Omentin	Omentin/Akt	Has anti-inflammatory action, reduces abdominal fat deposits	AT, Skeletal muscle	[117]

AMPK, AMP-activated protein kinase; TGF-β2, transforming growth factor-beta 2; FSTL1, follistatin-like 1; PPARα, peroxisome proliferator-activated receptor alpha; PRDM16, PR domain containing; SIRT1, sirtuin 1; GLUT-4, glucose transporter type 4. The plasma concentration of IL-6 is contingent upon exercise intensity and serve as a pivotal role in maintaining mitochondrial homeostasis while regulating thermogenesis in BAT [99,118]. FGF21, a key regulator of brown adipocyte differentiation, plays a major role in lipid and glucose homeostasis, insulin sensitivity, and mitochondrial biogenesis by upregulating PGC-1α in adipose tissue and skeletal muscle [111,119]. Furthermore, maternal exercise not only promotes metabolic dysfunction but also facilitates mitophagy and mitochondrial biogenesis. It enhances thermogenesis in offspring mice by increasing the expression of apelin, an exerkine, and adipokine. This effect mirrors the beneficial impact of exercise on fetal BAT generation and offspring metabolism, achieved through the heightened expression of PRDM16, a key transcription factor in brown adipogenesis [101,120]. Exercise-induced Metrnl is linked to increased fat oxidation and insulin sensitivity, reduced inflammation, modulation of mitochondrial homeostasis, and physiological effects associated with the browning of WAT [121,122]. BAIBA, a small-molecule myokine released from PGC-1α-expressing myocytes, promotes thermogenesis and induces WAT browning by increasing β-oxidation in hepatocytes by a PPARα-mediated mechanism in response to 8 weeks of treadmill exercise (50–60% maximal intensity). Additionally, BAIBA mitigates mitochondrial dysfunction and decreases cardiometabolic risk in individuals with obesity [107,123]. BDNF, a member of the neurotrophic factors, is expressed in skeletal muscle and functions as a myokine that regulates lipid oxidation and mitochondrial quality control by activating AMPK signaling in WAT in response to exercise [108,124]. Irisin, a PGC-1α-dependent myokine induced by exercise, plays a pivotal role in a brown-fat-like development program by stimulating UCP1 expression and mitochondrial biogenesis to counteract obesity [79]. Myostatin deficiency contributes to increased WAT browning, leading to heightened energy expenditure, improved insulin resistance, and stimulated mitochondrial biogenesis. This occurs through the regulation of irisin secretion via a novel miR-34a-dependent post-transcriptional mechanism [16,125] (Table 1).

## Data Availability

Data are available within the article.
